# Molecular autopsy in sudden cardiac death

**DOI:** 10.21542/gcsp.2023.8

**Published:** 2023-01-30

**Authors:** Oscar Campuzano, Georgia Sarquella-Brugada

**Affiliations:** 1 Medical Science Department, School of Medicine, Universitat de Girona, 17003 Girona, Spain; 2 Cardiovascular Genetics Center, University of Girona-IDIBGI, 17190 Girona, Spain; 3 Centro de Investigación Biomédica en Red, Enfermedades Cardiovasculares (CIBERCV), 28029 Madrid, Spain; 4 Pediatric Arrhythmias, Inherited Cardiac Diseases and Sudden Death Unit, Hospital Sant Joan de Déu, University of Barcelona, 08950 Barcelona, Spain; 5 European Reference Network for Rare, Low Prevalence and Complex Diseases of the Heart (ERN GUARD-Heart), 1105 AZ Amsterdam, The Netherlands; 6 Arrítmies pediàtriques, Cardiologia Genètica i Mort sobtada, Malalties Cardiovasculars en el Desenvolupament, Institut de Recerca Sant Joan de Déu, Esplugues de Llobregat, 08950 Barcelona, Spain

## Abstract

A post-mortem genetic analysis in the process of investigating a sudden death episode is known as ‘molecular autopsy’. It is usually performed in cases without a conclusive cause of death and after a comprehensive medico-legal autopsy. In these sudden unexplained death cases, an underlying inherited arrhythmogenic cardiac disease is the main suspected cause of death. The objective is to unravel a genetic diagnosis of the victim, but it also enables cascade genetic screening of the victim’s relatives. Early identification of a deleterious genetic alteration associated with an inherited arrhythmogenic disease may help to adopt preventive personalized measures to reduce risk of malignant arrhythmias and sudden death. It is important to remark that the first symptom of an inherited arrhythmogenic cardiac disease may the malignant arrhythmia and even sudden death. Next-generation sequencing allows a rapid and cost-effectives genetic analysis. Close interaction between the forensic scientist, pathologist, cardiologist, pediatric cardiologist and geneticist has allowed a progressive increase of genetic yield in recent years, identifying the pathogenic genetic alteration. However, large numbers of rare genetic alterations remain classified as having an ambiguous role, impeding a proper genetic interpretation and useful translation into both forensic and cardiological arena.

## Introduction

A complete medico-legal autopsy of a deceased person is termed inconclusive (‘negative autopsy’) in nearly 5% of all autopsies performed^1^. If the autopsy identifies no conclusive cause of death in an apparently healthy individual within an hour of symptom onset or within 24 h from the last time the person was seen alive, then these cases are defined as sudden unexplained death (SUD)^2^. In these SUD cases, an arrhythmogenic inherited disease (AID) is the most plausible cause of death^3^.

In population less than 40 years old, cardiomyopathies are main responsible for SUD, such as hypertrophic cardiomyopathy (HCM), dilated cardiomyopathy (DCM), and arrhythmogenic cardiomyopathy (ACM). However, in pediatric and young population^4^, channelopathies are main disorders responsible for SUD, such as long QT syndrome (LQTS), short QT syndrome (SQTS), Brugada syndrome (BrS), and catecholaminergic polymorphic ventricular tachycardia (CPVT)^5^.

Several studies highlight that between 10–20% of cases carry a definite pathogenic genetic alteration explaining the SUD, especially in the young population^5–10^. However, many molecular autopsies may be negative or inconclusive due to advanced Next Generation Sequencing (NGS) technologies identifying rare variants with unknown significance (VUS). Current clinical guidelines recommend molecular autopsy in SUD cases where cause of death is highly suspected to be due to AID^11–13^. Despite this fact, post-mortem genetic testing is not widely performed in most countries, mainly due to economic reasons or lack of protocol implementation in collection of post-mortem samples due to current legal restrictions involved with the sampling, storage and analysis of DNA^14^.

## Inherited Arrhythmogenic Diseases

### Long QT syndrome

This arrhythmogenic condition is characterized by prolongation of the QT interval on the ECG (QTc >460 ms in women and >450 ms in men). Clinical manifestations may range from asymptomatic to syncope or cardiac arrest due to *torsade de pointes* (TdP) in a structurally normal heart^13^. Unfortunately, sudden cardiac death (SCD) can be the first manifestation of the disease, especially in infants and the young population. Clinical events may be precipitated by specific triggers, including exercise, swimming, emotional stress or sudden loud noises^15^.

Concerning treatment, administration of beta-blocker is highly recommended because it decreases the risk of SCD, although it does not provide full protection^11^. Implantation of an ICD is mandatory for those patients who are survivors of an aborted SCD or in patients with a diagnosis of LQTS who experience recurrent syncopal events while on *β*-blocker therapy^16^. Left cardiac sympathetic denervation (LCSD) should be considered in high-risk patients with symptomatic LQTS in whom *β*-blockers are ineffective or not tolerated and in patients with recurrent appropriate ICD shocks despite maximum tolerated doses of *β*-blockers^17^.

**Figure 1.**
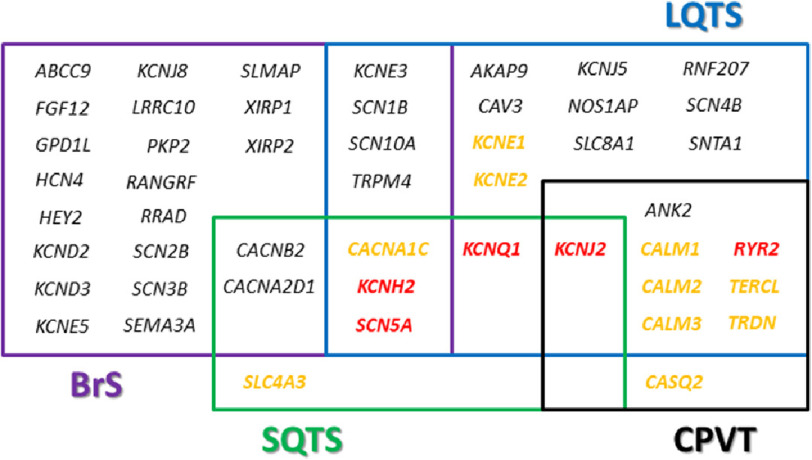
Genes currently associated with channelopathies. In red colour, five main genes associated with inherited channelopathies. In orange colour, genes strongly associated with inherited channelopathies. BrS: Brugada Syndrome; CPVT: Catecholaminergic Polymorphic Ventricular Tachycardia; LQTS: Long QY Syndrome; SQTS: Short QT Syndrome.

Currently, more than 1000 rare genetic alterations have been identified in almost 25 genes (Figure 1). An exhaustive genetic analysis of all reported genes identifies the cause of the disease in nearly 85% of cases. However, 80% of cases carry the genetic alteration in 3 main genes: *KCNQ1, KCNH2*, and *SCN5A*^18^. Hence, current guidelines recommend perform a genetic analysis of these 3 main genes in LQTS cases as the most cost-effective approach^11^. A negative autopsy of a sudden death at young age could be caused by this AID. Situation of death is crucial to unravel the cause of SUD since LQTS is related to exercise, swimming and even sleeping, depending on the gene mutation.

### Brugada syndrome

This AID is characterized by a coved-type ST-segment elevation in atypical right-bundle branch block in leads V1 to V3 (often referred to as a type-1 Brugada pattern) in the ECG^11^. The ECG pattern can be baseline or intermittent, and it can be unmasked during a drug test using class IC sodium channel-blockers. It is considered a disorder involving mainly young male adults (about 40 years old), and SCD typically occurring during sleep or at rest^13^.

Patients with BrS usually remain asymptomatic and modulating factors such as fever, exercise, or drugs may play a major role in the dynamic nature of the ECG pattern, increasing risk for SCD resulting from episodes of polymorphic ventricular tachyarrhythmias^19^. After surviving a cardiac arrest or the occurrence of syncope, the only treatment having any proven effect on the prevention of SCD is the ICD^20^. However, ICD implantation in asymptomatic patients is not free from controversy, especially in children^21^. Nowadays, more than 20 genes have been potentially associated to BrS (Figure 1) but pathogenic, or likely-pathogenic, variants have been reported only in *SCN5A* or associated sodium proteins^22^.

A comprehensive genetic analysis identifies the cause of disease in a 35% of cases, and nearly 30% of patients carry the genetic alteration in *SCN5A*. Hence, current guidelines recommend perform a genetic analysis of only *SCN5A* in BrS cases as the most cost-effective approach^11^. A negative autopsy of a young man who died at night could be caused by BrS.

### Short QT syndrome

This is a highly lethal arrhythmogenic disease characterized by a structurally normal heart with a shortened QT interval (QTc <340 ms), tall and peaked T waves, and poor rate adaptation of the QT interval^13^. In addition, this entity can be also diagnosed with a QTc interval of <360 ms with at least one of: (a) a definite pathogenic alteration; (b) a family history of SQTS; (c) a family history of SCD at age <40 years old, and (d) survival from a VT/VF episode in structural normal heart^11^.

Clinical manifestations may range from lack of symptoms to syncope and even SCD, sometimes the first symptom of the disease. An ICD is used to prevent SCD in high-risk patients. In patients with relatively benign phenotype, an individualized pharmacological measure may be use if ICD is not implanted, such as quinidine or hydroxyquinidine^23^. To date, no more than 50 pathogenic or likely pathogenic alterations have been associated with SQTS. These alterations are almost all located in 10 genes (Figure 1), mainly following an autosomal dominant pattern of inheritance^24^.

Comprehensive genetic analysis can identify the genetic alteration in 45% of clinically diagnosed cases, mostly located in 3 genes encoding potassium channels: *KCNQ1, KCNJ2*, and *KCNH*2^25^. Hence, current guidelines recommend genetic analysis of these 3 genes in SQTS cases as the most cost-effective approach^11^. SQTS usually occurs in the young population, and even infants, being considered main cause of death in first year of life (Sudden Infant Death Syndrome, SIDS)^26^. A negative autopsy of an infant who died suddenly could be caused by this malignant entity.

### Catecholaminergic polymorphic ventricular tachycardia (CPVT)

This is a lethal AID characterized by a bidirectional polymorphic ventricular tachycardia in a structurally normal heart^27^. The ECG is normal at rest (infrequently with bradycardia and U waves), and triggered exclusively by adrenergic stimulus, mainly exercise, excessive stress, or emotion^13^.

Administration of beta-blockers is the first therapeutic approach, but flecainide had added a significant improvement in controlling arrhythmias in these patients. Left cardiac sympathetic denervation, performed by experts, is a very useful tool for patients not controlled by medication or intolerant to beta-blockers. Therefore, ICD only remains indicated for patients with aborted SCD and incompletely controlled VT, despite a full therapeutic arsenal^28^.

Concerning genetics, more than 200 pathogenic, or likely-pathogenic, alterations have been reported so far in no more than 10 genes (Figure 1), mainly following an autosomic dominant pattern of inheritance. A comprehensive genetic analysis of all reported genes explains 65% of CPVT cases, but nearly 55% are due to genetic alterations located in the *RyR2* gene^29^. Hence, current guidelines recommend perform a genetic analysis of only this gene in CPVT cases as the most cost-effective approach^11^.

CPVT is a main cause of unexplained SCD in young population(<40 years old and predominately males)^30^, mainly in adolescents and children before 10 years old, and not rarely the first symptom of the disease^31^. Hence, a negative autopsy in a young patient who died suddenly during an adrenergic situation could be due to CPVT.

## Genetic Analysis

The first studies on molecular autopsy performed a post-mortem genetic analysis of a limited number of genes using Sanger technology^6–8,10,32^, according to guidelines available at the time^12^.

Nowadays the high fidelity of Sanger sequencing, despite it being slow and expensive, remains the gold-standard and is used mainly to validate new NGS findings (mainly insertion/deletion sequences), cascade segregation of variants in relatives, and amplification of regions not covered by NGS technology (mainly regions of the genome rich in cytosine and guanine nucleotides).

It is widely accepted that NGS technology is the approach that should be used in molecular autopsy, thanks to its high genetic yield at low-effective cost with limited amounts of DNA and in a reduced time^33,34^. Current NGS approaches include personalized panels of genes associated with a specific disease, whole exome sequencing (WES), and even whole genome sequencing (WGS), all at similar cost.

For genetic diagnosis, only panels have been used regularly. Both WES and WGS approaches are used mainly for research proposes. It is important to remark that some hospitals and centres of genetic diagnosis use WES approach but a final report is only performed focused on a list of genes associated with the suspected or diagnosed disease, in the same way that occurs using a panel^35–41^.

In comparison to the Sanger approach, the genetic yield of WES increases the detection of variants in young SUD cohorts, mainly due to more genes being analyzed, but when it comes to rare variants, there is generally no conclusive role in AID. Diagnostic yields have increased nearly 35% thanks to the combination of molecular autopsy and clinical evaluation^42^.

The current challenge is not in the technical approach, but rather a proper genetic interpretation of the data obtained after genetic analysis and useful clinical translation. Current guidelines of the American College of Medical Genetics and Genomics (ACMG) recommend the use of standard terminology to classify variants: ‘pathogenic’, ‘likely pathogenic’, ‘likely benign’, ‘benign’, and ‘variant of unknown significance’ (VUS)^43^. ACMG allows an exhaustive classification of variants, but a lack of available data leads to an ambiguous role in genetic alterations^43–45^.

Before clinical translation, a group of experts in each area should discuss the role of variants included in the final report since it may alter the personalized therapeutic measures adopted with the deceased’s relatives, especially if a variant remains as VUS^46^.

## Recommendations

In 2008, members of Trans-Tasman Response AGAinst sudden Death in the Young (TRAGADY), also endorsed by the Royal College of Pathologists of Australasia and officially endorsed by the National Heart Foundation of New Zealand, proposed a guide to standardize the practice of autopsies in SUD in young people and pay appropriate attention to family members.

Several guidelines/recommendations have been published in this way. In 2020, Stiles et al published a consensus statement focused on SUD cases and victim’s relatives. The authors strongly recommended genetic testing if AID is suspected as a cause of SUD. A comprehensive autopsy should include collection and storage of tissue suitable for genetic analysis. Where the autopsy suggests a possible genetic cause, or no cause, and the heart is normal, referral to a multidisciplinary team for further investigation is indicated.

Cascade genetic testing and counseling should be previously discussed with families to ensure that risks, benefits, results, and the clinical significance of genetic testing are considered. In addition, a detailed personal and family history is essential in a SUD case, with attention to sentinel symptoms during life, such as syncope or seizures, witness accounts, premorbid investigations, and inspection of any cardiac rhythm monitoring around the time of death. Clinical assessment of family members should be also performed, including physical examination, ECGs, cardiac imaging, ambulatory monitoring, and provocative testing with multidisciplinary team supervision. Follow-up and periodic re-evaluation are important and are directed by initial findings^47^.

Recently, Wilde et al published current expert consensus statement on the state of genetic testing for AID. Authors recommend retain sample of SUD case for molecular autopsy is an AID is identified during autopsy or suspected. Clinical assessment in family members should be perfumed and, if a genetic alteration identified in victim, cascade genetic analysis should be performed in relatives^13^.

## Conclusions

Molecular autopsy is a crucial tool to unravel the cause of a SUD case. In these cases, AID is the most plausible cause of death, therefore the victim’s relatives may also be at risk due to genetic origin. Improvement in cost-effective NGS technologies allow an increasing genetic yield, especially in the infant, pediatric, and young population. However, lack of functional data impedes a proper genetic interpretation and a large percentage of genetic alterations identified after a comprehensive genetic analysis remain as VUS. In these cases, transition into clinical practice should be done with caution, with close collaboration between forensic scientist, pathologist, cardiologist, pediatric cardiologist and geneticist.

## Conflict of Interest

All authors have reported that they have no relationships relevant to the contents of this paper to disclose.

## Acknowledgement/Funding

This work was supported by Obra Social “La Caixa Foundation” (LCF/PR/GN19/50320002). Co-funded by Instituto de Salud Carlos III (FIS PI21/00094). CIBERCV is an initiative of the ISCIII, Spanish Ministry of Economy and Competitiveness. Funders had no role in study design, data collection, data analysis, interpretation, or writing of the report.

## Ethics approval

The study was approved by the ethics committee of Hospital Sant Joan de Déu and followed the World Medical Association Declaration of Helsinki. Written informed consent was obtained both from parents of all patients and from all relatives included in the study.
